# Unveiling the in vitro activity of extracted *Euphorbia trigona* via Supercritical Fluid Extraction against pathogenic yeasts, obesity, cancer, and its wound healing properties

**DOI:** 10.1186/s40643-025-00855-y

**Published:** 2025-04-04

**Authors:** Abdulrahman S. Bazaid, Naif K. Binsaleh, Heba Barnawi, Bandar Alharbi, Ahmed Alsolami, Samy Selim, Soad K. Al Jaouni, Amna A. Saddiq, Magdah Ganash, Tarek M. Abdelghany, Husam Qanash

**Affiliations:** 1 Department of Medical Laboratory Science, College of Applied Medical Sciences, University of Ha’il, Hail, 55476 Saudi Arabia; 2 Medical and Diagnostic Research Center, University of Ha’il, Hail, 55473 Saudi Arabia; 3 Department of Internal Medicine, College of Medicine, University of Ha’il, Hail, 55476 Saudi Arabia; 4 Department of Clinical Laboratory Sciences, College of Applied Medical Sciences, Jouf University, Sakaka, 72388 Saudi Arabia; 5 Department of Hematology/Oncology, Yousef Abdulatif Jameel Scientific Chair of Prophetic Medicine Application, Faculty of Medicine, King Abdulaziz University, Jeddah, 21589 Saudi Arabia; 6 Department of Biological Sciences, Faculty of Science, University of Jeddah, Jeddah, Saudi Arabia; 7 Department of Biology Science, College of Science, King Abdulaziz University, Jeddah, 21589 Saudi Arabia; 8 Botany and Microbiology Department, Faculty of Science, Al-Azhar University, Cairo, 11725 Egypt

**Keywords:** Rosmarinic acid, Molecular docking, Yeasts, *Euphorbia trigona*, Cancer, Wound healing

## Abstract

Natural products of plant origin are being explored as safe alternatives for illness management. Their extraction processes play a crucial role in determining their phytochemical and pharmacological properties. In this context, *Euphorbia trigona* was extracted using Supercritical Fluid Extraction with CO_2_ (SFE-CO_2_) at two operating temperatures: 20 °C and 40 °C. Phytochemical characterization was performed via HPLC, along with anti-yeast evaluation using the well diffusion method, anticancer assessment using the MTT assay, wound healing analysis via the scratch assay, and anti-obesity evaluation through the lipase assay of the *E. trigona* extract. The results indicated that SFE-CO_2_ at 40 °C extracted a greater quantity (0.198 g) of *E. trigona* than SFE-CO_2_ at 20 °C (0.156 g). Several compounds, such as rosmarinic acid, gallic acid, daidzein, ellagic acid, naringenin, and ferulic acid, were identified at high concentrations of 10,034.29, 1,800.33, 750.22, 748.11, 462.15, and 207.05 µg/mL, respectively, in the *E. trigona* extract obtained using SFE-CO_2_ at 40 °C, compared to the extract obtained using SFE-CO_2_ at 20 °C. High inhibition zones of 24 ± 1.5, 24 ± 0.5, and 23 ± 0.33 mm were recorded against *C. albicans*, *C. tropicalis*, and *G. candidum*, respectively, using the extract from SFE-CO_2_ at 40 °C, compared to the inhibition zones of 24 ± 1.5, 24 ± 0.5, and 23 ± 0.33 mm obtained from the extract using SFE-CO_2_ at 20 °C. Moreover, the extract from SFE-CO_2_ at 40 °C exhibited lower MIC and MFC values against the tested yeasts compared to the efficacy of the extract from SFE-CO_2_ at 20 °C. The ultrastructure of the examined yeasts was severely affected by the extract from SFE-CO_2_ at 40 °C. A lower IC_50_ (98.87 ± 1.26 µg/mL) was recorded for the extract from SFE-CO_2_ at 40 °C compared to the IC_50_ (333.87 ± 1.8 µg/mL) of the extract from SFE-CO_2_ at 20 °C against cancer cells (A431). The wound closure level was 84.08% using the extract from SFE-CO_2_ at 40 °C, while it was 71.27% using the extract from SFE-CO_2_ at 20 °C. Lipase was inhibited by the extract obtained via SFE-CO_2_ at 40 °C and 20 °C, with IC_50_ values of 15.77 and 28.14 µg/mL, respectively. Molecular docking indicated that rosmarinic acid is a suitable inhibitor for the tested yeasts.

**Graphical Abstract:**

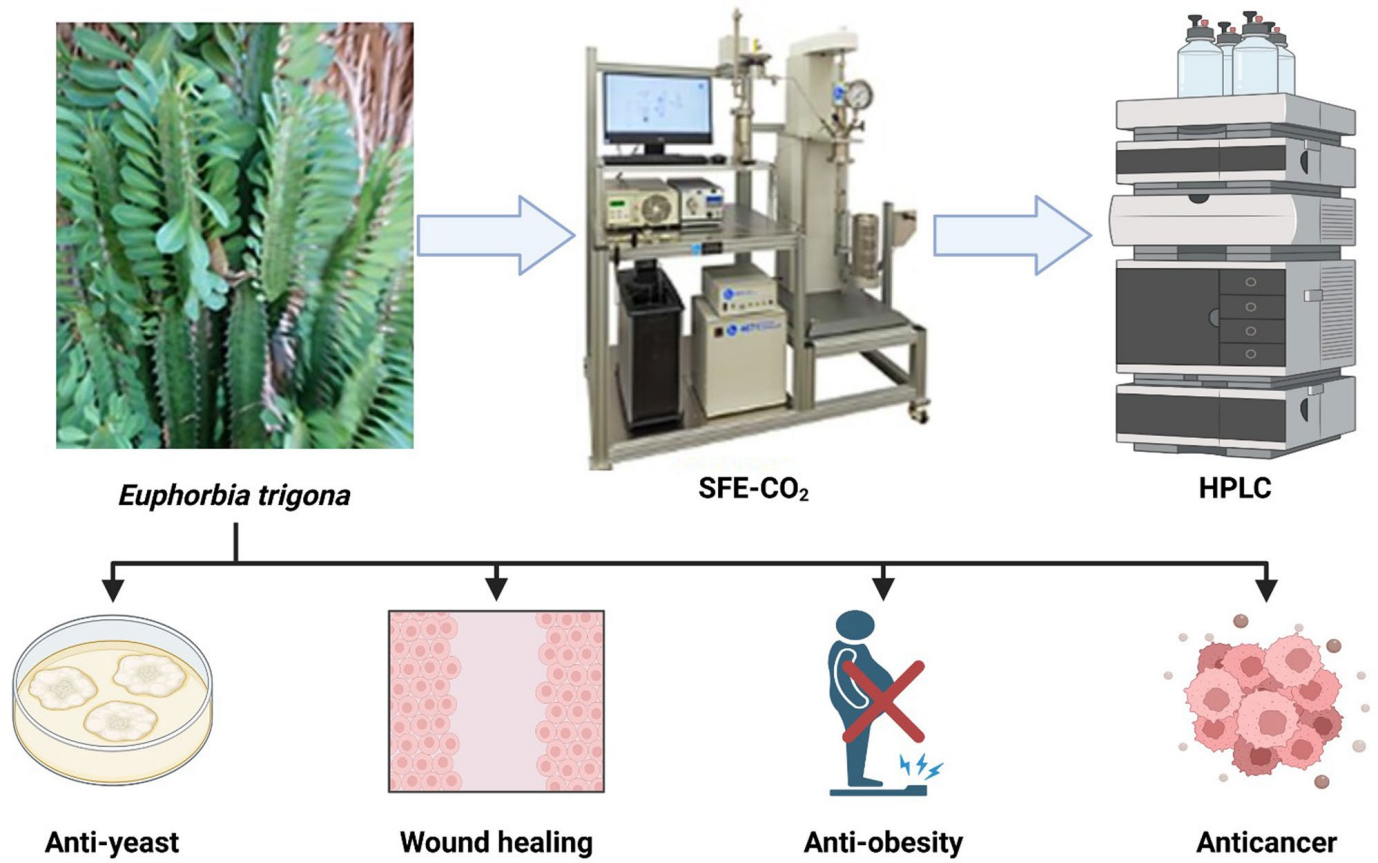

**Supplementary Information:**

The online version contains supplementary material available at 10.1186/s40643-025-00855-y.

## Introduction

Several investigations have been conducted on the application of natural sources of plant or microbial origin for the management of numerous illnesses in humans and animals (Abdelghany et al. 2019; Yahya et al. 2022; Al-Rajhi and Abdelghany 2023a, b). The biological activities of plant ingredients are associated with their active compounds, such as phenols, flavonoids, alkaloids, saponins, terpenoids, vitamins, and other metabolites (Abdelghany et al. 2021; Bogolitsyn et al. 2019). These groups of compounds can occur in various organs of the plant, including seeds, grains, leaves, fruit, stems, and roots (Qanash et al. 2022; Al-Rajhi et al. 2023b, c; Alawlaqi et al. 2023).

Different investigations have documented that members of the *Euphorbiaceae* family contain tannins, flavonoids, and terpenes (- Anju and Rameshkumar 2022; Ourhzif et al. 2022; Zaghlol et al. 2024). The current investigation focuses on one member of the *Euphorbiaceae* family, namely *Euphorbia trigona*. Various illness disorders were managed in vitro using *E. trigona* extract, including infections caused by pathogenic yeasts, cancer, obesity, and wound healing. The emergence of yeasts as a pathogenic risk presents an important challenge to community health, particularly among immunocompromised patients and in intensive care settings. As mentioned in other studies, *E. trigona* extract possesses numerous biological applications, such as antifungal and antioxidant properties (Hammadi et al. 2021). Based on prior investigations (Deenen and Prüfer 2011), the development of *Botrytis cinerea*, *Fusarium graminearum*, and *Aspergillus niger*, which are filamentous fungi, was inhibited by *E. trigona* extract. Additionally, bacteria such as *Pseudomonas aeruginosa* and *Proteus mirabilis* were inhibited by the latex of *E. trigona* extract (Nashikkar et al. 2011).

Until now, cancer has been considered one of the most hazardous illnesses, with high mortality rates globally. According to a literature review by Jiménez-González et al. (Jiménez-González et al. 2024), the number of cancer cases is expected to increase and reach 24 million by 2040. In fact, conventional chemical therapies for cancer patients lack both selectivity and optimal efficacy. For this reason, several investigations have focused on utilizing natural resources to address this problem (Al-Rajhi and Abdelghany 2023a, b; Selim et al. 2024). The anticancer activity of *E. trigona* has been documented against human hepatoma and colon adenocarcinoma (El-Hawary et al. 2020). Anju and Rameshkumar (- Anju and Rameshkumar 2022) reported that *E. trigona* exhibited non-toxic and insignificant toxic effects on cardiac myoblasts (normal cells) and cervical cancer cell lines, respectively, at a concentration of 100 µg/mL. Wounds are recognized as one of the main issues in developing nations, frequently leading to serious consequences that increase the cost of treatment. Therefore, the search for compounds with wound healing properties has become essential to address this issue. In a literature review on wound healing management using *Euphorbia* plants, Alsaffar et al. (Alsaffar et al. 2021) stated that this group of plants is considered a promising agent for wound healing.

There are several extraction methods for plant materials that depend on chemical solvents. However, in recent decades, numerous investigators have focused on the Supercritical Fluid Extraction with CO_2_ (SFE-CO_2_) method, which offers a high yield of extracts with many active constituents while preserving the characteristics important for medicinal applications. This technique can be employed for the extraction of polyphenols from plant materials and greatly simplifies extraction procedures, minimizing the extraction period due to its low viscosity and high density. Furthermore, the degradation of extracted materials is avoided with SFE, as it operates in the absence of air and light (Almehayawi et al. 2024). Several advantages of SFE-CO_2_ utilization were reported such as useless the organic solvents, reproducibility, stability and suitability because of adaptable power of solvation (pressure and temperature). CO_2_ is the greatest generally used supercritical fluid because it’s valuable properties. CO_2_ works as non-inflammable, harmless, and economical gas. Moreover, CO_2_ can be effortlessly withdrawn from the extract at applied atmospheric conditions during the extraction process (Bogolitsyn et al. 2019). To address issues related to maximizing the quantity and activity of extracted compounds from natural sources, our investigation aims to utilize the SFE method to extract from *E. trigona* and evaluate its activity against pathogenic yeasts, cancer, obesity, and wound healing, in addition to conducting molecular docking of the main detected flavonoid against the tested yeasts.

## Materials and methods

### Source of plant and their yield extract via supercritical CO2 extraction (SFE-CO2)

Aerial parts, including succulent stems and leaves of *Euphorbia trigona*, were collected from a garden and authenticated by botanist Professor Mohamed Amin. The collected parts of *E. trigona* were washed with water and then dried in the shade at 50 °C for 3 days. After that, they were ground to a fine powder. *E. trigona* powder (3 g) can be extracted using the SFE-CO_2_ process, which utilizes the unique properties of supercritical fluids. In this process, carbon dioxide is heated to its supercritical form under pressure, at which point it exhibits characteristics of both a gas and a liquid. This allows it to efficiently extract desired chemicals from plants without leaving any solvent residue. The supercritical CO_2_ extraction apparatus, which typically consists of a high-pressure vessel, a pump, a heat exchanger, and a collection vessel, must first be assembled. The equipment needs to be constructed to withstand the high heat and pressures involved in the process. The *E. trigona* powder is placed inside the extraction vessel, which is then sealed to contain CO_2_ during the extraction. The pump is used to introduce CO_2_ into the system, and the pressure is gradually increased to reach the supercritical state. The extraction process was performed under two different temperature conditions: 20 °C and 40 °C, while other factors remained constant throughout the extraction, including pressure (3650 PSI), static extraction time (20 min), and dynamic extraction time (40 min).

### Analysis of flavonoids and phenolic acids in *E. trigona* extracts by HPLC

The high-performance liquid chromatography (HPLC) analyses were conducted using a Shimadzu Prominence HPLC system (Kyoto, Japan). The mobile phase consisted of acetonitrile (first solvent) and a 1% (v/v) aqueous acetic acid solution (second solvent). The injection volume was maintained at 20 µL, the thermostatically controlled column was set to 28 °C, and the flow rate was set at 0.7 mL/min. To perform gradient elution, the ratio of the first solvent to the second solvent was varied. The gradient elution was adjusted linearly from 10 to 63% of the first solvent over 11 min, with a 10-minute hold at 63%. After 26 min, the elution was increased to 90%. After 31 min, the composition returned to the starting point (first solvent: second solvent = 10:90), and the system was allowed to run for an additional five minutes before the next sample was injected. A photodiode array UV detector operating at 272 nm was used to analyze the HPLC chromatograms (Qanash et al. 2023a, b, c).

### Anti-yeast activity of *E. trigona* extract

Yeast inhibition by the *E. trigona* extracts was measured using the well-plate agar diffusion assay against the following *Candida* species: *C. glabrata* (RCMB 027016), *C. albicans* (ATCC 10231), and *C. tropicalis* (ATCC 10243). The yeasts were standardized to the 0.5 McFarland scale and then placed into plates seeded with sterile, melted Sabouraud dextrose medium. After the mixture set, cups (6-mm radius) were removed from the agar layer using a sterile cork borer. An automated microliter pipette was used to place 100 µL of each *E. trigona* extract (20 µg/mL) into each cup. The inoculated plates were kept at 5 °C for 2 h to allow for the diffusion of the extract across the agar layer and were subsequently incubated for 48 h at 27 °C. Wells containing fluconazole served as a control. The inhibition zones were measured at the end of the incubation period (Abdelghany et al. 2021).

### Minimum concentration of *E. trigona* extract for yeast Inhibition and as a fungicidal agent

The twofold broth dilution technique was used to assess the minimum inhibitory concentration (MIC) (French 2006). The extract was then serially diluted twofold from the initial dose of 125 µg/mL and mixed with 2 mL of sterile broth. A tube without extract served as the negative control. Next, 0.5 mL of an 18-hour-old culture of each yeast, previously adjusted to 0.5 McFarland (10^6^ CFU/mL), was added to each tube and thoroughly mixed using a micropipette. The tubes were placed in an incubator at 35 °C for 24 h, after which they were observed for growth, indicated by turbidity. The MIC was determined as the test tube with the lowest dilution that showed no visible growth without the aid of a microscope. After identifying the MIC tubes that exhibited no cloudiness, they were streaked onto Sabouraud’s dextrose agar plates and left to incubate at 35 °C for one day. The minimum fungicidal concentration (MFC) was defined as the lowest quantity that prevented any observable growth during the incubation period.

### Anticancer potential of *E. trigona* extract

The anticancer activity of *E. trigona* extract was evaluated on the A431 cell line (human skin carcinoma) and the WI38 cell line (normal human fetal lung fibroblasts), which were provided by Vacsera, Giza, Egypt. Cells (5 × 10^4^ cells/well) were cultured in 96-well plates using RPMI 1640 medium supplemented with 10% heat-inactivated fetal bovine serum (FBS) and antibiotics (penicillin and streptomycin at doses of 100 U/mL and 100 µg/mL, respectively). The cells were incubated for 24 h in a humidified atmosphere with 5% CO_2_, after which the excess medium (100 µL) was removed. Various doses of the plant extract (ranging from 31.25 to 1000 µg/mL) were added to the microplate, and the plates were incubated for an additional day at 37 °C. Afterward, 10 µL of 3-(4,5-dimethylthiazol-2-yl)-2,5-diphenyl-2 H-tetrazolium bromide (MTT) at a stock concentration of 5 mg/mL was added, followed by incubation of the microplate at 37 °C for another 4 hours. For each well, 150 µL of DMSO (dimethyl sulfoxide) was added to dissolve the formazan crystals. The absorbance was measured at 490 nm using a microplate reader Unexposed cells were incubated in the same conditions as the treated cells and were used as a control. The morphological features of the cells exposed to the plant extract were imaged using a light microscope (phase contrast) (Nikon Corporation) in bright field.

### Scratch wound closure for assaying the wound healing properties of plant extract

Fibronectin extracellular matrix substrate (10 µg/mL) was coated onto a multi-well plate and incubated for two hours at 37 °C. After the unbound extracellular matrix was removed, phosphate-buffered saline (PBS) was used as a wash. The detached cells were removed from the tissue culture plate using trypsin. The cells were then placed on a scratched scan plate, where they were allowed to multiply and form an identical monolayer. The monolayer that was generated was gently scraped with the tip of a pipette. After scraping, the layer containing cells was lightly rinsed to eliminate any detached cells. Finally, a freshly prepared medium containing verified cells was used. The plate was kept in a cell culture incubator set at 37 °C for up to 48 h. After this incubation period, PBS was used to wash the cell layer. Subsequently, the cells were fixed via 3.7% paraformaldehyde for 15 min. The cells were stained with 1% crystal violet in ethanol for 10 min. The resulting cells were examined using a phase-contrast microscope (Martinotti and Ranzato 2020). The analysis was computed as follows using the subsequent equations:

$${\rm{Migration}}\,{\rm{rate}}\left( {{\rm{MR}}} \right) = {\eqalign{& {\rm{Initial}}\,{\rm{width}}\,{\rm{of}}\,{\rm{wound}}\,{\rm{}}\left( {{\rm{\mu m}}} \right) \cr& - {\rm{Final}}\,{\rm{width}}\,{\rm{of}}\,{\rm{wound}}\left( {{\rm{\mu m}}} \right) \cr} \over {{\rm{Assay}}\,{\rm{requred}}\,{\rm{time}}\left( {{\rm{hours}}} \right)}}{\rm{}} \times 100$$

$$\eqalign{{\rm{Clouser}}\,{\rm{of}}\,{\rm{wound}}\,\left( {\rm{\% }} \right){\rm{}} = & {\eqalign{& {\rm{Intial}}\,{\rm{area}}\,{\rm{of}}\,{\rm{wound}} \cr& - {\rm{Area}}\,{\rm{of}}\,{\rm{Wound}}\,{\rm{after}}\,\left( {\rm{n}} \right){\rm{}}\,{\rm{hours}} \cr} \over {{\rm{Intial}}\,{\rm{area}}\,{\rm{of}}\,{\rm{wound}}}} \cr& \times 100 \cr} $$

$$\:\text{A}\text{r}\text{e}\text{a}\:\text{d}\text{i}\text{f}\text{f}\text{e}\text{r}\text{e}\text{n}\text{c}\text{e}\:\left(\text{\%}\right)=\text{I}\text{n}\text{t}\text{i}\text{a}\text{l}\:\text{a}\text{r}\text{e}\text{a}-\text{F}\text{i}\text{n}\text{a}\text{l}\:\text{a}\text{r}\text{e}\text{a}$$

### Anti-obesity activity of *E. trigona* extract via lipase Inhibition

According to Kim et al. (Kim et al. 2010), the inhibition of lipase activity by varying doses of both plant extract and the standard control (Orlistat) (7.8 to 1000 µg/mL dissolved in DMSO) was detected using the substrate 4-nitrophenyl butyrate. Potassium phosphate buffer (pH 7.0, 0.1 mM) was employed to prepare a stock solution of lipase (5 mg/mL). The reaction mixture consisted of 5 µL of 15 mM 4-nitrophenyl butyrate, 35 µL of enzyme, 220 µL of potassium phosphate buffer, and 5 µL of plant extract, respectively. All these components were preserved for 10 min at 37 ºC. The released quantity of 4-nitrophenol from the reaction was estimated at 405 nm. Lipase inhibition was measured from the subsequent equation:

$$\:\text{L}\text{i}\text{p}\text{a}\text{s}\text{e}\:\text{i}\text{n}\text{h}\text{i}\text{b}\text{i}\text{t}\text{i}\text{o}\text{n}\:\left(\text{\%}\right)=\frac{\text{A}\text{b}\text{s}.\text{c}\text{o}\text{n}\text{t}\text{r}\text{o}\text{l}-\text{A}\text{b}\text{s}.\text{s}\text{a}\text{m}\text{p}\text{l}\text{e}}{\text{A}\text{b}\text{s}.\text{c}\text{o}\text{n}\text{t}\text{r}\text{o}\text{l}}\:\times\:100$$

### Experimental molecular Docking

Molecular modeling is critical in the rational development and discovery of drug methodologies. Molecular docking, a trusted and adaptable in silico technology that employs a novel computational approach, was used to anticipate the binding behavior of each protein, and ligands were chosen. All docking calculations were performed using the Molecular Operating Environment program.

We conducted molecular docking studies of rosmarinic acid against the crystal structures of *C. albicans* (PDB ID: 1ZAP), *C. tropicalis* (PDB ID: 6ZD6), and *G. candidum* (PDB ID: 6ISV) proteins. These proteins involved in virulence (SAP2), sterol biosynthesis (CYP51), and metabolism (oxidoreductase), respectively. These proteins were chosen due to their importance in fungal survival, pathogenicity, and antifungal resistance, making them attractive drug targets. Rosmarinic acid was obtained from the PubChem website and prepared using energy minimization and partial charge optimization. The proteins used were retrieved from the RCSB website (www.rcsb.org). Following the removal of the water molecules surrounding the protein, each target was constructed using corrections, 3D hydrogenation, and energy minimization. Docking was performed on the rigid receptor atoms, and rosmarinic acid (the ligand) was inserted into the binding site using the triangle matcher technique. London dG was employed as a scoring function, with GBVI/WSA dG procedures used for rescoring. Dummy atoms were generated using the acquired alpha spheres. The docked ligand’s leading conformations were determined by comparing Root Mean Square Deviation (RMSD) levels, binding energies, and binding modes to the essential residues. If the algorithm can determine the optimal pose under a preselected RMSD level of the identified conformation (often 1.5 or 2 Å, based on ligand size), the approach is referred to as “validated.

### Statistical analysis

The findings obtained were analyzed using SPSS version 15.0 (SPSS Inc., Chicago, IL, USA). The ± standard deviation (SD) was calculated from the mean values of three repetitions of each result.

## Result and discussion

### Extraction yield of *E. trigona* and their phytochemical characterization

**Fig. 1:**
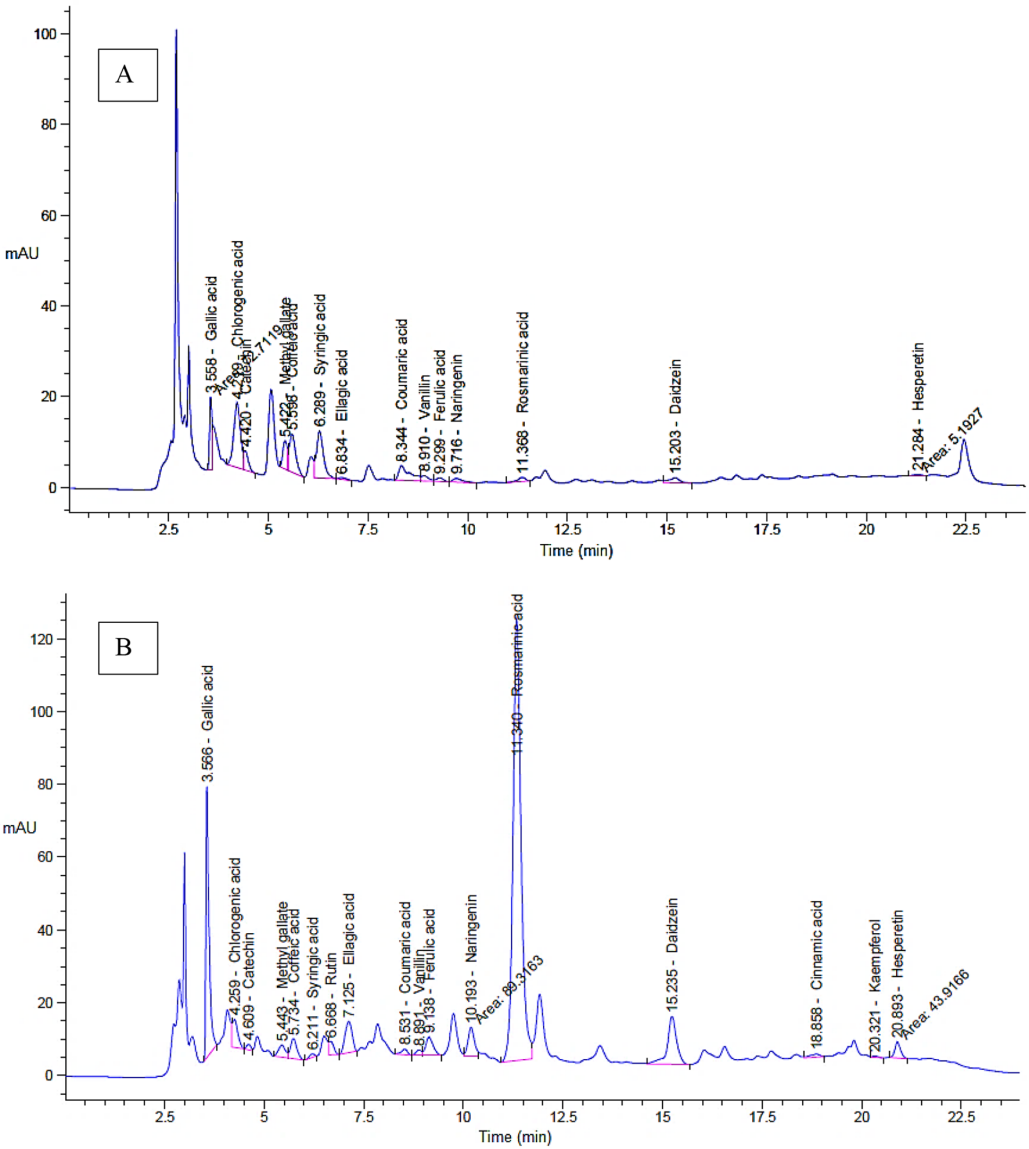
HPLC Chromatograms of separated compounds from *E. trigona* extract via SFE-CO_2_ at 20 °C (**A**) and *E. trigona* extract via SFE-CO_2_ at 40 °C (**B**)

Our specific paper employed the extracted *E. trigona* via SFE-CO_2_ under different conditions for chemical characterization using HPLC and tested its biological functions. The extraction yield of *E. trigona* via SFE-CO_2_ was affected by temperature; it was 0.198 g when SFE-CO_2_ operated at 40 °C, while it was 0.156 g at 20 °C (Supplementary 1).The extraction was performed at 60 °C but the yield of extract was less than that at 20 and 40 °C (data not tabulated), therefore the analysis of extract and their biological activities were achieved using the extract at 20 and 40 °C. This result indicates that temperature is a critical factor in the extraction process. Our results agree with those of other researchers who employed SFE-CO_2_ at different temperatures (Silva et al. 2017). Several characteristics of the extract were attributed to the analysis conducted by HPLC (Fig. 1A and b). For instance, high concentrations of phenols and flavonoids were detected in the extract of *E. trigona* via SFE-CO_2_ at 40 °C, including rosmarinic acid (10,034.29 µg/mL), gallic acid (1,800.33 µg/mL), daidzein (750.22 µg/mL), ellagic acid (748.11 µg/mL), naringenin (462.15 µg/mL), and ferulic acid (207.05 µg/mL). Furthermore, some phenols and flavonoids, such as rutin (271.88 µg/mL), cinnamic acid (13.48 µg/mL), and kaempferol (13.04 µg/mL), were detected only in the extract obtained via SFE-CO_2_ at 40 °C and not at 20 °C (Table 1). The appearance or disappearance of certain phenols and flavonoids indicates that extraction conditions may affect the content of active ingredients in the extract. On the other hand, the concentrations of chlorogenic acid, catechin, and syringic acid were higher when compared to their concentrations in the extract obtained via SFE-CO_2_ at 20 °C. According to the standard compounds injected in HPLC, pyrocatechol and quercetin were not detected in the extract. The edited review article by Magozwi et al. (Magozwi et al. 2021) mentioned that *Euphorbia* spp. flavonoids play vital roles, including antioxidants, antibacterial, antitumor, anti-inflammatory, antimalarial, and anti-hepatitis functions. Through HPLC analysis, some of the detected compounds in *E. trigona* were also found in *E. tirucalli*, including gallic acid, catechin, caffeic acid, chlorogenic acid, ellagic acid, and ferulic acid (Araújo et al. 2014). HPLC analysis of different species, including *E. eriophora*, *E. aleppica*, *E. macroclada*, *E. grisophylla*, *E. craspedia*, *E. seguieriana*, *E. denticulata*, and *E. fistulosa*, characterized various compounds; among those that matched our study were rutin, chrysophanic acid, and hesperidin (Yener et al. 2019). Soliman et al. (Soliman et al. 2021) detected some compounds associated with flavonoids and phenols in *E. cuneata*, such as pyrogallol, kaempferol, rutin, and acids including coumaric, syringic, caffeic, gallic, and ferulic.

**Table 1:** Identified compounds in extract of *E. trigona* at two temperature values via SFE-CO_2_

Identified constituent	SFE-CO_2_ at 20 °C			SFE-CO_2_ at 40 °C		
	Area (%)	Retention time	Concentration (µg/mL)	Area (%)	Retention time	Concentration (µg/mL)
Gallic acid	10.76	3.56	226.76	13.97	3.57	1800.33
Chlorogenic acid	24.18	4.22	877.46	2.50	4.26	554.41
Catechin	5.05	4.42	309.56	0.42	4.61	158.78
Methyl gallate	7.12	5.42	100.07	1.44	5.44	123.94
Caffeic acid	13.74	5.60	282.15	2.16	5.73	288.37
Syringic acid	18.44	6.29	333.38	0.30	6.21	33.03
Pyro catechol	0.00	6.43	0.00	0.00	6.43	0.00
Rutin	0.00	6.65	0.00	1.05	6.67	271.88
Ellagic acid	0.70	6.83	23.47	3.65	7.13	748.11
Coumaric acid	9.16	8.34	85.53	0.54	8.53	30.93
Vanillin	2.06	8.91	20.40	0.47	8.89	28.27
Ferulic acid	1.33	9.30	30.21	2.23	9.14	207.05
Naringenin	1.86	9.72	45.60	3.09	10.19	462.15
Rosmarinic acid	1.80	11.37	50.37	58.7	11.34	10034.29
Daidzein	3.04	15.20	50.73	7.35	15.24	750.22
Querectin	0.00	16.94	0.00	0.00	16.94	0.00
Cinnamic acid	0.00	18.90	0.00	0.46	18.86	13.48
Kaempferol	0.00	20.35	0.00	0.11	20.32	13.04
Hesperetin	0.77	21.28	38.52	1.52	20.90	118.69

### Anti-yeast potential of *E. trigona* with MIC, MFC and ultrastructure

**Fig. 2:**
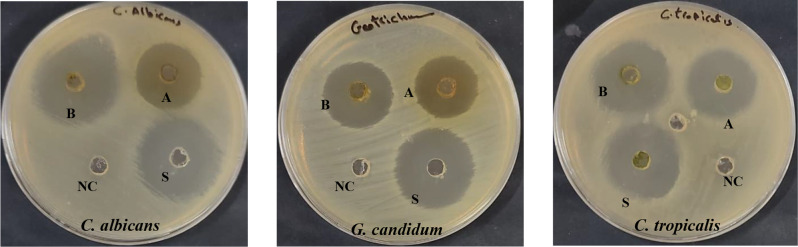
Anti-yeast properties of *E. trigona* extract at different temperatures of extraction. SFE-CO_2_ at 20 °C (**A**), SFE-CO_2_ at 40 °C (**B**), standard antifungal (S), and Negative control (NC)

The illustrated findings (Fig. 2; Table 2) indicated that the *E. trigona* extract obtained via SFE-CO_2_ at 40 °C was more effective than the extract obtained via SFE-CO_2_ at 20 °C when tested against the examined yeasts. The findings reflect the existence of inhibition zones of 33 ± 0.5, 27 ± 0.33, and 26 ± 0.66 mm associated with *C. albicans*, *C. tropicalis*, and *G. candidum*, respectively, using the extract obtained at 40 °C, compared to inhibition zones of 24 ± 1.5, 24 ± 0.5, and 23 ± 0.33 mm for the extract obtained at 20 °C. The standard antifungal exhibited more significant inhibition zones than the employed extract. An earlier investigation by Kirbag et al. (Kirbag et al. 2013) also documented the anti-yeast activity of *Euphorbia* species against *C. glabrata*, *Epidermophyton* sp., *C. albicans*, and *C. tropicalis*; moreover, *C. tropicalis* was the most susceptible fungus to all used *Euphorbia* extracts. The *Euphorbia* species studied in the current investigation inhibited the growth of the mycotoxigenic fungi *F. graminearum* and *A. niger*, as previously mentioned (Deenen and Prüfer 2011). Anti-yeast activity was also achieved using other species; *C. tropicalis*, for instance, was inhibited by various extracts (using different solvents) of *E. cuneata*, with inhibition zones ranging from 13.5 to 22.5 mm based on the applied solvent (Yener et al. 2019). The MIC and MFC values of the extract obtained via SFE-CO_2_ at 20 °C were higher than those of the extract obtained at 40 °C for all examined yeasts, confirming the efficacy of the extract obtained via SFE-CO_2_ at 40 °C. Rosmarinic acid was the highest identified phenol in *E. trigona* extract according to HPLC analysis; it prevented the development of *C. albicans* and *A. parasiticus*, respectively, at MIC values of 7.82 and 15.62 µg/mL. Additionally, it caused morphological deformation in the examined fungi (Vidya et al. 2024). In another investigation, the mechanism of rosmarinic acid’s activity against fungi was described in relation to different medical isolates of *Candida*, such as *C. krusei*, *C. albicans*, *C. glabrata*, *C. parapsilosis*, and *C. tropicalis* (Ivanov et al. 2022), with the MIC of rosmarinic acid ranging from 100 to 200 µg/mL. In the same context, changes in membrane integrity, a decline in mitochondrial function, and minor inhibition of protease activity were observed in the isolates exposed to rosmarinic acid.

**Table 2:** Anti-yeast properties of *E. trigona* extract at different conditions of extraction with Estimation of MIC and MFC

Investigated yeast	Zone of inhibition (mm)			MIC (µg/mL)		MFC (µg/mL)	
	SFE-CO_2_ at 20 °C	SFE-CO_2_ at 40 °C	Control	SFE-CO_2_ at 20 °C	SFE-CO_2_ at 40 °C	SFE-CO_2_ at 20 °C	SFE-CO_2_ at 40 °C
*C. albicans*	24 ± 1.5	33 ± 0.5	28 ± 0.5	15.62	3.9	31.25	15.62
*C. tropicalis*	24 ± 0.5	27 ± 0.33	28 ± 0.25	31.25	7.8	31.25	15.62
*G. candidum*	23 ± 0.33	26 ± 0.66	30 ± 0.5	31.25	15.62	62.5	31.25

**Fig. 3:**
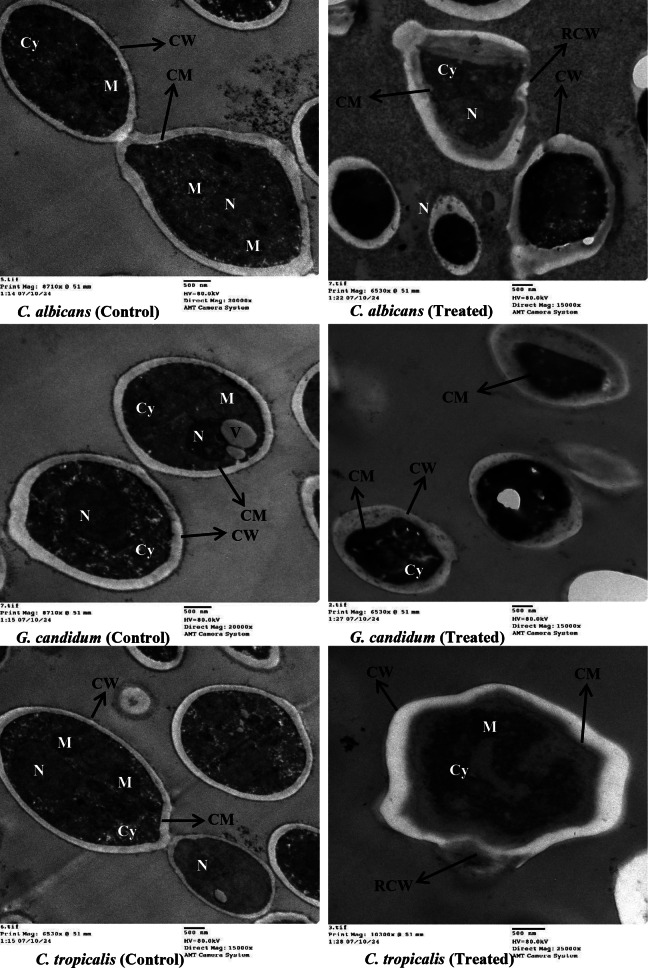
Image analysis of treated yeasts using TEM. Cell wall (CW), cytoplasm (Cy), cell membrane (CM), rupture cell wall (RCW), mitochondria (M), Vacuole (V) and Nucleus (N)

The examined yeasts, utilizing Transmission electron microscopy (TEM) after being exposed to *E. trigona* extract through SFE-CO_2_ at 40 °C, were visualized (Fig. 3). It is clear that all three yeast species, *C. albicans*, *C. tropicalis*, and *G. candidum*, were affected by the *E. trigona* extract. The untreated yeasts exhibited clear and distinct cell walls, cell membranes, nuclei, mitochondria, and cytoplasm, while the treated yeasts displayed irregular and ruptured cell walls, along with the collapse of cell membranes distant from the cell walls, as observed in both *C. albicans* and *C. tropicalis*. In treated *G. candidum*, severe cytoplasmic shrinking was noted. There are currently no studies on the effect of *E. trigona* extract on yeast ultrastructure via TEM. A previous report utilized Scanning Electron Microscopy (SEM) to examine *C. albicans* and another species, *E. hirta*; their leaf extracts showed cell wall cracks that may be due to the loss of metabolic functions in the yeast cells (Rajeh et al. 2010). Additionally, using TEM, morphological alterations and cell lysis, with complete collapse of treated *C. albicans* by *E. hirta* leaf extracts, were documented. In contrast, the untreated *C. albicans* displayed a normal shape with a consistent central density in the nucleus and numerous components of the endomembrane system, alongside the presence of a regular and intact cell wall (Basma et al. 2011). The present findings from our study utilizing TEM document the fungicidal potential of *E. trigona* extract. The observed changes, particularly in the cell walls and cell membranes, may indicate disruptions in cell permeability.

### Anticancer properties of *E. trigona* extract

**Fig. 4:**
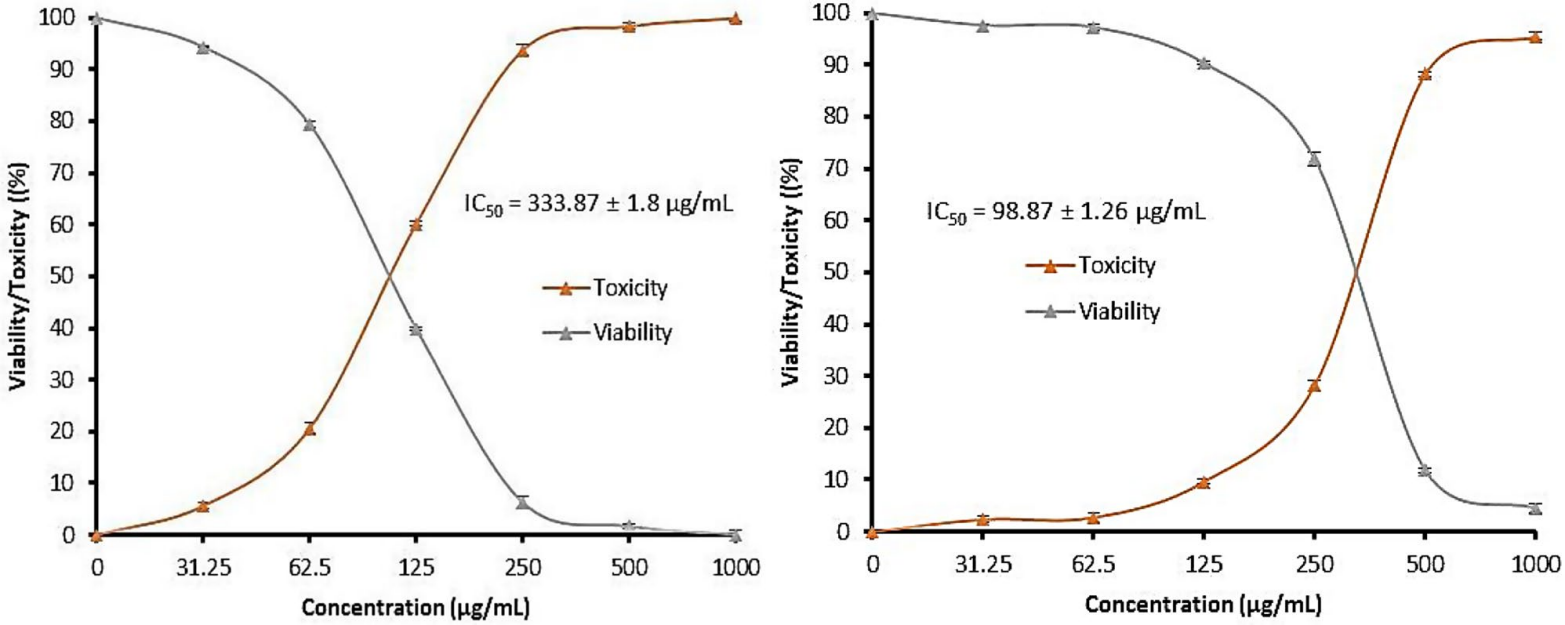
Anticancer properties of *E. trigona* extract at different temperatures of extraction against A431 cells. SFE-CO_2_ at 20 °C (left figure), SFE-CO_2_ at 40 °C (right figure)

The anticancer potential of *E. trigona* extract against A431 cells with varying levels based on concentration and extraction conditions was reported (Fig. 4). When using *E. trigona* extract via SFE-CO_2_ at 20 °C, the anticancer activity was lower than that observed with the extract at 40 °C across all examined concentrations. For instance, 79.36%, 6.30%, and 0.13% toxicity were recorded with the extract at 20 °C, while 97.29%, 71.82%, and 4.53% toxicity were noted with the extract at 40 °C, at concentrations of 1000, 125, and 31.25 µg/mL, respectively. All these findings were documented through the estimation of IC_50_ values (333.87 ± 1.8 µg/mL for the extract at 20 °C and 98.87 ± 1.26 µg/mL for the extract at 40 °C) (Fig. 4). From the calculation of IC_50_ values for the extracts at 20 °C, it is evident that these extracts do not possess a cytotoxic effect on normal cells (WI38), where the IC_50_ values were 615.13 ± 5.5 µg/mL and 579.87 ± 3.66 µg/mL, respectively (data not tabulated). A431 cell death was induced by the extract, particularly at high doses (250 to 1000 µg/mL) (Supplementary 2 &3) Furthermore, shrinkage was observed in the cell line, and the cells became detached and rounded. Low doses (31.25 to 125 µg/mL) of the extracts did not show any alterations in cell shape compared to unexposed cells (control). Anticancer activities were associated with some but not all species of *Euphorbia*. For instance, the development of human hepatoma cells was repressed by *E. lunulata* (Gao et al. 2021). Seham et al. (Seham et al. 2021) reported the anticancer potential of *E. lactea* against breast adenocarcinoma (MCF-7) and hepatoma (HepG2) cell lines, while *E. trigona* extract was effective on MCF-7 and Caco-2 cancerous cell lines; however, the tested cancer cells were not affected by *E. ingens*, *E. horrida*, and *E. tirucalli*. The anti-proliferative potential of *E. trigona* against four types of cancer cells, including H116, HeLa (cervical cancer cell line), A549, and HL-60, was reported by Villanueva et al. (Villanueva et al. 2015). Anju and Rameshkumar (Anju and Rameshkumar 2022) demonstrated that *E. trigona* extract exhibited no toxicity and insignificant toxicity against the normal cell line H9C2 (cardiac myoblasts) and HeLa, respectively, up to a dose of 100 ​µg/mL.

### Wound healing properties of *E. trigona* extract

**Fig. 5:**
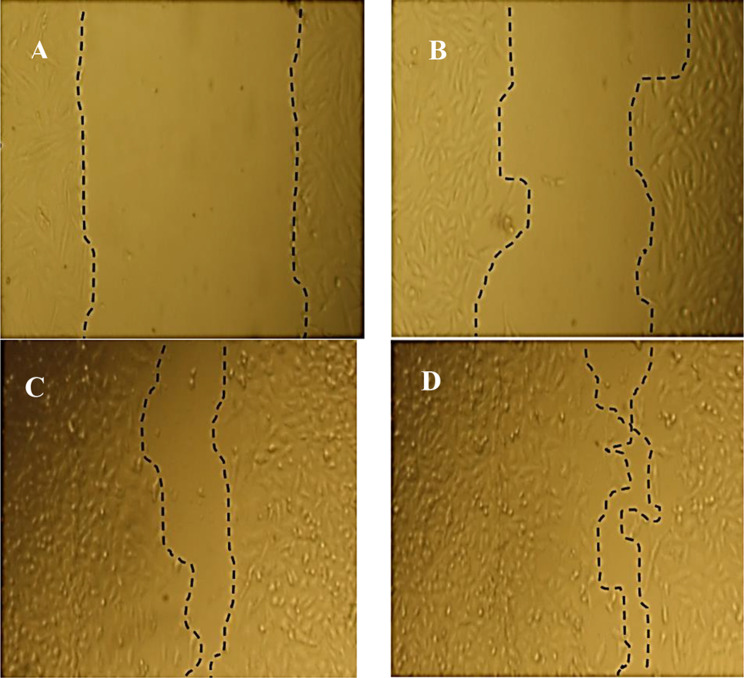
Effect of *E. trigona* extract on the wound healing via Scratch assay. HFB4 cells at 0 h (**A**) and 48 h (**B**) without treatment compared to treated cells by extract via SFE-CO_2_ at 20 °C (**C**) and SFE-CO_2_ at 40 °C (**D**) after 48 h

In the current study, it is clear that *E. trigona* extract plays a critical role in the healing of wounds, particularly when the extract is used via SFE-CO_2_ operated at 40 °C (Supplementary 4 and Fig. 5). Accelerated wound healing was observed with the extract at this temperature, where wound closure percentage was 84.08% compared to treatment using the extract via SFE-CO_2_ operated at 20 °C (71.27%) or to untreated cells (26.18%). Other key metrics documenting the wound healing included area difference and re-epithelialization, which increased in the treated cells to 1,010,433% and 19.67 μm, respectively, with the extract via SFE-CO_2_ operated at 40 °C. Based on recent investigations (Al-Rajhi et al. 2024), the healing of wounds may be induced by a reduction in tissue inflammation and oxidative stress. This suggests that the extract possesses antioxidant and anti-inflammatory properties. According to Zulkefli et al. (Zulkefli et al. 2023), numerous studies have shown that flavonoids play an essential role in wound healing due to their effects on angiogenesis and re-epithelialization. The plant extract investigated in this paper was rich in various phenols and flavonoids, particularly in the extract obtained via SFE-CO_2_ operated at 40 °C; therefore, wound healing was faster with this extract than with the extract operated at 20 °C. Chaniad et al. (Chaniad et al. 2020) found that catechin was effective in wound healing. In the present study, rutin was detected only in the extract via SFE-CO_2_ operated at 40 °C, and this flavonoid possesses wound-healing properties (Hou et al. 2021). Additionally, previous reports indicated that gallic acid accelerated wound healing by protecting the skin from oxidative stress.

### Anti-obesity properties of *E. trigona* via lipase Inhibition

**Fig. 6:**
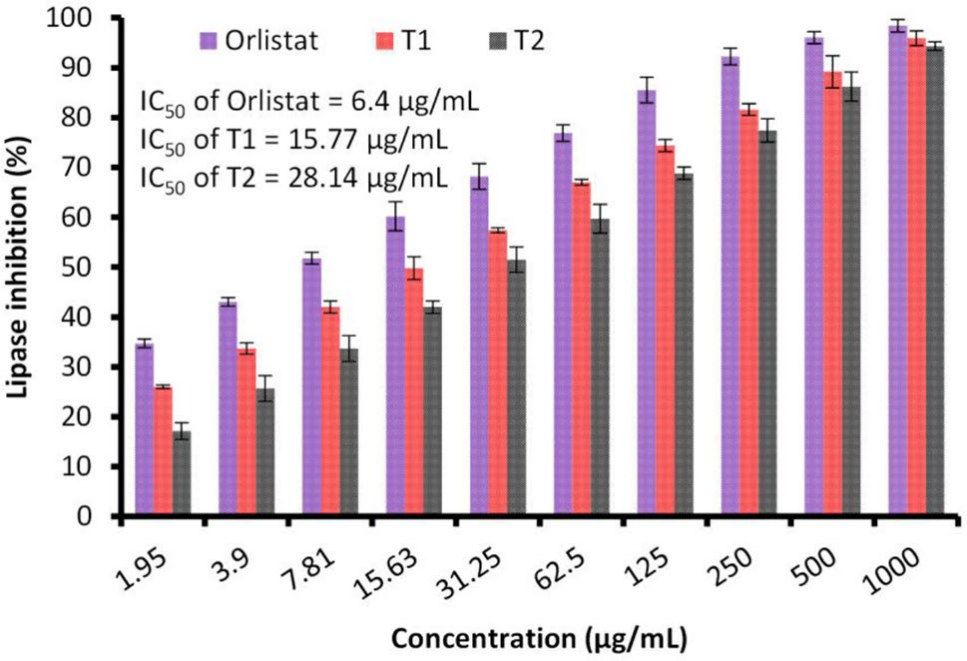
Effect of *E. trigona* extract and orlistat on lipase. Extract via SFE-CO_2_ at 20 °C (T2) and SFE-CO_2_ at 40 °C (T1) after 48 h

Inhibition of lipase was documented under the influence of *E. trigona* extract via SFE-CO_2_ operated at 20 °C and 40 °C (Fig. 6). A decline in lipase activity was observed when exposed to high concentrations of *E. trigona* extract. As noted in the current findings, the extract via SFE-CO_2_ operated at 40 °C revealed greater lipase inhibition than the extract via SFE-CO_2_ operated at 20 °C across all tested doses. An IC_50_ of 15.77 µg/mL was obtained for the extract via SFE-CO_2_ operated at 40 °C, compared to an IC_50_ of 28.14 µg/mL for the extract via SFE-CO_2_ operated at 20 °C, while Orlistat (the standard drug) has an IC_50_ of 6.4 µg/mL for lipase inhibition. Lipase inhibition is one of the most commonly investigated mechanisms for evaluating the potential effects of newly discovered or developed drugs for obesity management (D’Costa et al. 2024). Although various therapeutic strategies for treating obesity have been researched, few are considered safe, and most have negative side effects. Therefore, an alternative is to discover anti-obesity substances from natural sources. The presence of rutin, rosmarinic acid, and chlorogenic acid in *E. trigona* has been extensively confirmed through HPLC analysis. Previous research indicates that these compounds play a central role in regulating obesity (Meng et al. 2013). Rosmarinic acid has been shown to play a vital role in suppressing tissue inflammation and preventing the accumulation of excess lipids in adipocytes (Vasileva et al. 2021). Furthermore, molecular analysis has confirmed that rosmarinic acid has the potential to help manage obesity.

### Molecular Docking interaction

Molecular docking is a type of bioinformatics modeling that combines two or more constituents to form a stable adduct. This technique produces many possible conjugate structures, which are then graded and categorized using the software’s scoring functions. The data obtained from the docking technique can be utilized to determine a ligand’s binding energy, stability, and free energy (Qanash et al. 2022; Al-Rajhi et al. 2024). Molecular docking was conducted on *C. albicans* (PDB ID: 1ZAP), *C. tropicalis* (PDB ID: 6ZD6), and *G. candidum* (PDB ID: 6ISV) proteins to better understand the enzyme-ligand interaction and establish the optimal orientation of the ligand within the active site. The results presented in Supplementary 5 demonstrate a significant alignment between the docking data and the experimental findings. Supplementary 6 outlines the amino acids involved in interaction formation, along with a comprehensive list of all ligand interactions, including hydrogen bonds and hydrophobic interactions. Rosmarinic acid was selected for molecular docking because represent the main detected compound with the highest concentration 10034.29 µg/mL *E. trigona* extract via SFE-CO_2_ at 40 °C. Docking studies revealed that the inhibitor rosmarinic acid can effectively engage with certain amino acids based on their active regions. The tested molecule also appeared to have adequate RMSD values.

**Fig. 7:**
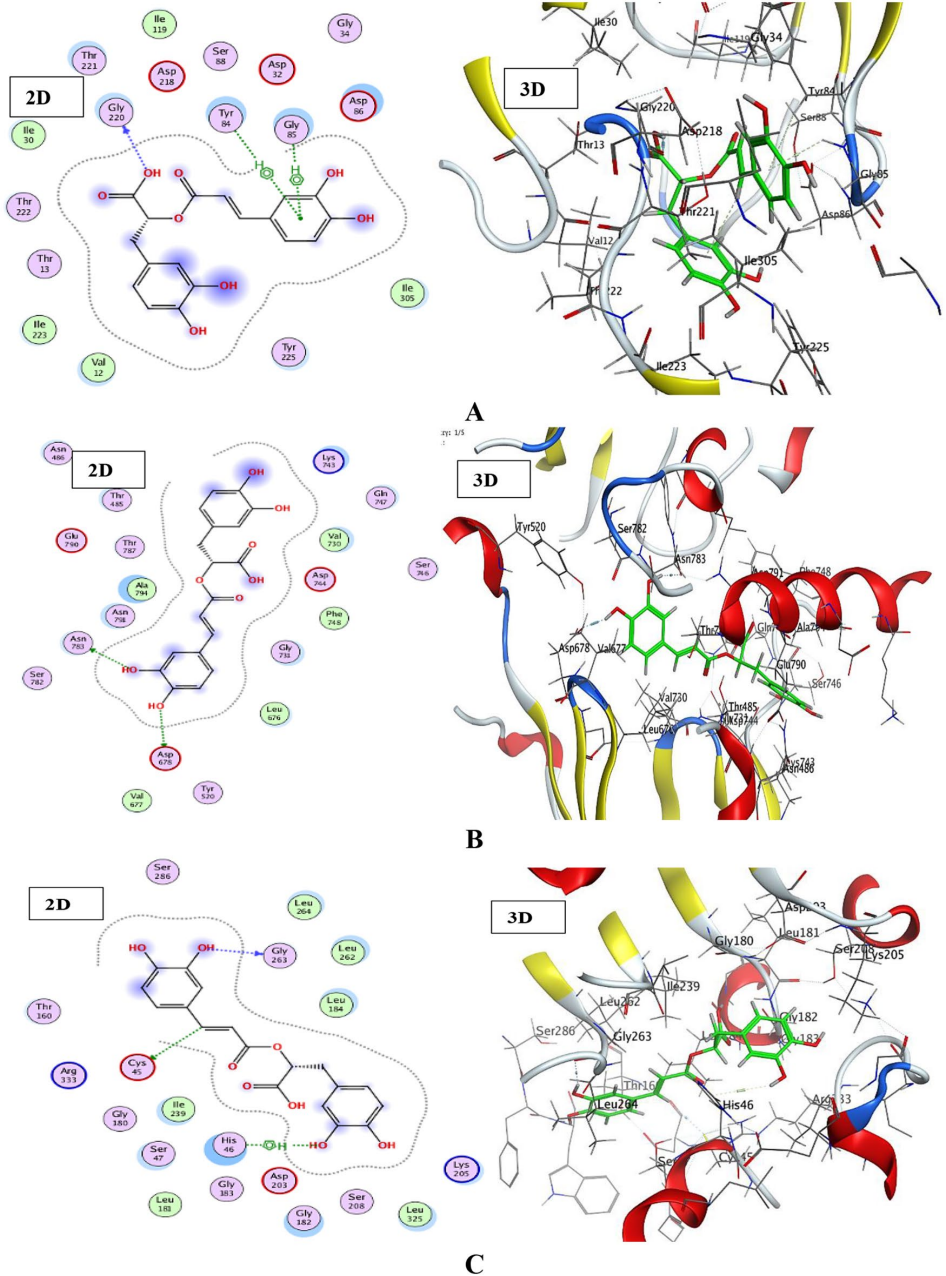
2D and 3D graphs show the interaction among rosmarinic acid and active sites of *C. albicans* (PDB ID: 1ZAP) protein (**A**), rosmarinic acid and active sites of *C. tropicalis* (PDB ID: 6ZD6) protein (**B**), and rosmarinic acid and active sites of *G. candidum* (PDB ID: 6ISV) protein (**C**)

The ligand used to inhibit (1ZAP), (6ZD6), and (6ISV) developed binding values of -6.15839, -6.79996, and − 6.8224 kcal/mol, respectively. In addition, various interactions were observed between the investigated ligand and receptors, such as H-donor and Pi-H interactions. Rosmarinic acid bonded to *Candida albicans* (PDB ID: 1ZAP) through amino acid residues GLY 220, TYR 84, and GLY 85 via O 17 and a six-membered ring in the ligand while interacting with the protein receptors in the pocket of *C. tropicalis* (PDB ID: 6ZD6) through ASP 678 and ASN 783 by O 35 and O 37 ligand atoms. Furthermore, the inhibitor ligand with *G. candidum* (PDB ID: 6ISV) demonstrated direct H-donor links with active site residues CYS 45 and GLY 263 through C 24 and O 37 atoms, as well as an H-pi bond between the O 39 atom and the five-membered ring of HIS 46 amino acid residues. The best-fitted 2D and 3D poses chosen by the examined compound are reported (Fig. 7). The interactions between rosmarinic acid and yeast protein receptors are illustrated via symbolic keys (Supplementary 7).

The interaction of molecular docking has been performed in numerous studies to predict or discover the activity of compounds towards target proteins in organisms (Al-Rajhi et al. 2022, 2023b, c; Al-Rajhi and Abdelghany 2023a, b; Qanash et al. 2023a, b, c). The main detected compounds, namely ellagic acids and chlorogenic acid from the Henna plant, were docked with target proteins of both *G. candidum* and *C. albicans* (Alsalamah et al. 2023), where the resulting binding scores were − 5.69876 kcal/mol and − 7.84379 kcal/mol for the docking of chlorogenic acid with *C. albicans* (4YDE) and *G. candidum* (4ZZT), respectively, whereas the scores were − 4.5145 kcal/mol and − 6.18615 kcal/mol for the docking of ellagic acid with *C. albicans* (4YDE) and *G. candidum* (4ZZT), respectively.

## Conclusion

In the current study, it is possible to conclude that SFE-CO_2_ can alter the biological activities of *E. trigona*. SFE-CO_2_ increased the yield of the *E. trigona* extract as well as the concentrations of the most detected flavonoids and phenolic compounds, based on the results of HPLC analysis. Anti-yeast activity was observed against *C. albicans*, *C. tropicalis*, and *G. candidum* using *E. trigona* extract, particularly from the extract processed at 40 °C using SFE-CO_2_. The *E. trigona* extract obtained via SFE-CO_2_ at 40 °C exhibited greater anticancer activity than the extract obtained at 20 °C. Additionally, obesity was regulated through lipase inhibition using *E. trigona* extract. Significant results were recorded using the *E. trigona* extract processed with SFE-CO_2_ at 40 °C in wound healing. Molecular docking of rosmarinic acid (the main compound detected in the *E. trigona* extract) with the active proteins in the examined yeasts was performed and supported the anti-yeast activity of *E. trigona* extract.

## Electronic supplementary material

Below is the link to the electronic supplementary material.

Supplementary Material 1

## Data Availability

Not applicable.
