# Supplementary material for: Unveiling the in vitro activity of extracted Euphorbia trigona via Supercritical Fluid Extraction against pathogenic yeasts, obesity, cancer, and its wound healing properties
Source: Bioresour Bioprocess. 2025 Apr 4;12(1):28. doi: 10.1186/s40643-025-00855-y (PMC11971087; doi:10.1186/s40643-025-00855-y)
Supplement: Supplementary file 1 — Supplementary Material 1 [file 40643_2025_855_MOESM1_ESM.docx]

**Supplementary 1. Yield extract of E. trigona via SFE-CO_2_ at different temperatures, constant pressure and constant extraction static and dynamic times.**

| **Temperature (°C)** | **Pressure (PSI)** | **Static time of extraction (min)** | **Dynamic time of extraction (min)** | **Quantity (g)** | **Extract quantity (g)** |
| --- | --- | --- | --- | --- | --- |
| 20 | 3650 | 20 min | 40 | 3.0 | 0.156 |
| 40 | 3650 | 20 min | 40 | 3.0 | 0.198 |


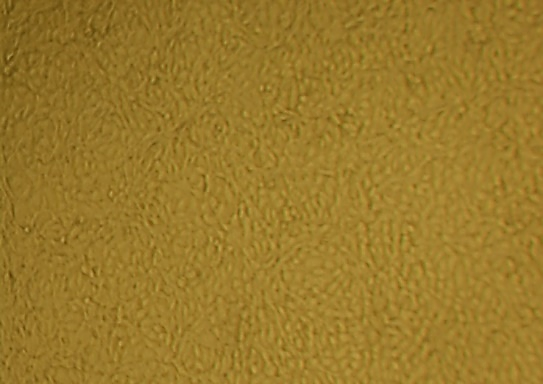


**Control**

**1000 µg/mL**

**500 µg/mL**

**250 µg/mL**

**125 µg/mL**

**31.25 µg/mL**

**62.5 µg/mL**

**Supplementary 2. Morphological changes in exposed A431 cells to different concentrations of E. trigona extract using SFE-CO_2_ at 20 °C.**

**Supplementary 3. Morphological changes in exposed A431 cells to different concentrations of E. trigona extract using SFE-CO_2_ at 20 °C.**

**Supplementary 4. *In vitro,* healing of exposed wounds to E. trigona extracts via SFE-CO_2_ at 20 °C and SFE-CO_2_ at 40 °C.**

| **Treatment** | **At 0 h** | | **At 28 h** | | **RM um** | **Wound Closure % µm** | **Area Difference %** |
| --- | --- | --- | --- | --- | --- | --- | --- |
|  | **Area** | **Width** | **Area** | **Width** |  |  |  |
| Exposed cells to extract via SFE-CO_2_ at 40 °C | 1142.06 | 1219828 | 312.03 | 333272.5 | 19.67 | 84.08 | 1010433 |
|  | 1106.03 | 1190095 | 100.18 | 107794.4 |  |  |  |
|  | 1072.12 | 1147169 | 140.06 | 149861.3 |  |  |  |
|  | 1078.05 | 1153560 | 96.75 | 103523.8 |  |  |  |
|  | 1196.54 | 1277907 | 230.22 | 245871.8 |  |  |  |
|  | 1142.00 | 1221996 | 194.04 | 207633 |  |  |  |
|  | Mean | | | |  |  |  |
|  | 1122.8 | 1201759 | 178.88 | 191326.1 |  |  |  |
| Exposed cells to extract via SFE-CO_2_ at 20 °C | 1142.06 | 1219828 | 398.01 | 425106 | 16.67 | 71.27 | 856527 |
|  | 1106.03 | 1190095 | 412.08 | 443398.8 |  |  |  |
|  | 1072.12 | 1147169 | 422.12 | 451667.1 |  |  |  |
|  | 1078.05 | 1153560 | 422.30 | 451884.1 |  |  |  |
|  | 1196.54 | 1277907 | 142.00 | 151656 |  |  |  |
|  | 1142.00 | 1221996 | 138.01 | 147681.5 |  |  |  |
|  | Mean | | | |  |  |  |
|  | 1122.8 | 1201759 | 322.42 | 345232.2 |  |  |  |
| Control  HFB4 cells | 1142.06 | 1219828 | 982.00 | 1048868 | 6.12 | 26.18 | 314565.2 |
|  | 1106.03 | 1190095 | 742.04 | 798443.5 |  |  |  |
|  | 1072.12 | 1147169 | 768.09 | 821862.1 |  |  |  |
|  | 1078.05 | 1153560 | 728.00 | 778997.4 |  |  |  |
|  | 1196.54 | 1277907 | 918.01 | 980433.6 |  |  |  |
|  | 1142.00 | 1221996 | 836.0 | 894559.3 |  |  |  |
|  | Mean | | | |  |  |  |
|  | 1122.8 | 1201759 | 829.0252 | 887194.1 |  |  |  |

**Supplementary** **5.** Docking scores and energies of rosmarinic acid with Crystal structure of C. albicans (PDB ID: 1ZAP), C. tropicalis (PDB ID: 6ZD6), and G. candidum (PDB ID: 6ISV) receptors.

| **Mol** | **Protein** | **S** | **rmsd_refine** | **E_conf** | **E_place** | **E_score1** | **E_refine** | **E_score2** |
| --- | --- | --- | --- | --- | --- | --- | --- | --- |
| Rosmarinic acid | 1ZAP | -6.15839 | 3.381634 | -32.9683 | -80.5616 | -12.0718 | -31.1964 | -6.15839 |
| Rosmarinic acid | 1ZAP | -6.15567 | 2.718567 | -34.2867 | -88.9901 | -12.6102 | -31.3619 | -6.15567 |
| Rosmarinic acid | 1ZAP | -6.0153 | 2.225292 | -36.474 | -106.256 | -11.5697 | -31.7875 | -6.0153 |
| Rosmarinic acid | 1ZAP | -5.98006 | 1.679687 | -39.5727 | -77.4506 | -11.926 | -34.9946 | -5.98006 |
| Rosmarinic acid | 1ZAP | -5.97516 | 2.293432 | -38.7321 | -90.0014 | -12.4549 | -30.9408 | -5.97516 |
| Rosmarinic acid | 6ZD6 | -6.79996 | 1.61781 | -35.6314 | -100.025 | -11.8875 | -39.6861 | -6.79996 |
| Rosmarinic acid | 6ZD6 | -6.78277 | 2.029022 | -28.2313 | -85.348 | -12.183 | -39.2188 | -6.78277 |
| Rosmarinic acid | 6ZD6 | -6.61033 | 1.858217 | -38.8954 | -96.8103 | -11.6986 | -34.6557 | -6.61033 |
| Rosmarinic acid | 6ZD6 | -6.45225 | 1.754059 | -35.3265 | -120.915 | -12.1372 | -36.6602 | -6.45225 |
| Rosmarinic acid | 6ZD6 | -6.45035 | 2.229034 | -31.849 | -104.129 | -12.3572 | -30.2835 | -6.45035 |
| Rosmarinic acid | 6ISV | -6.8224 | 1.170835 | -36.4616 | -74.3233 | -13.0308 | -31.1422 | -6.8224 |
| Rosmarinic acid | 6ISV | -6.76436 | 2.475577 | -30.1791 | -66.0753 | -12.3767 | -33.1459 | -6.76436 |
| Rosmarinic acid | 6ISV | -6.73617 | 2.460847 | -41.8284 | -86.0005 | -13.1703 | -37.5135 | -6.73617 |
| Rosmarinic acid | 6ISV | -6.73391 | 2.243541 | -38.4045 | -73.9749 | -12.3934 | -33.2932 | -6.73391 |
| Rosmarinic acid | 6ISV | -6.72827 | 1.43336 | -31.9283 | -118.242 | -12.4189 | -31.919 | -6.72827 |

**Supplementary** **6.** Interaction of Rosmarinic acid with Crystal structure of C. albicans (PDB ID: 1ZAP), C. tropicalis (PDB ID: 6ZD6), and G. candidum (PDB ID: 6ISV) receptors.

| **Mol** | **Protein** | **Ligand** | **Receptor** | **Interaction** | **Distance** | **E (kcal/mol)** |
| --- | --- | --- | --- | --- | --- | --- |
| Rosmarinic acid | 1ZAP | O 17 | O GLY 220 (A) | H-donor | 2.88 | -4.2 |
|  |  | 6-ring | CB TYR 84 (A) | pi-H | 4.68 | -0.5 |
|  |  | 6-ring | N GLY 85 (A) | pi-H | 3.56 | -0.7 |
| Rosmarinic acid | 6ZD6 | O 35 | OD1 ASP 678 (A) | H-donor | 2.84 | -4.5 |
|  |  | O 37 | OD1 ASN 783 (A) | H-donor | 2.77 | -0.7 |
| Rosmarinic acid | 6ISV | C 24 | SG CYS 45 (A) | H-donor | 3.78 | -1.3 |
|  |  | O 37 | O GLY 263 (A) | H-donor | 3.25 | -0.6 |
|  |  | O 39 | 5-ring HIS 46 (A) | H-pi | 4.40 | -2.6 |


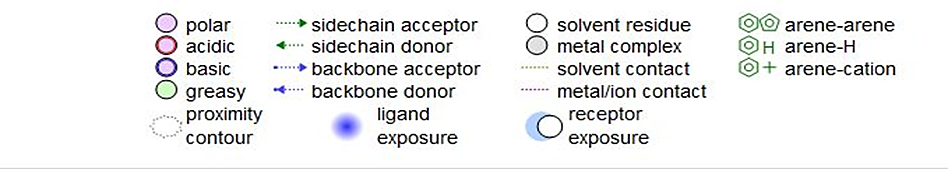


**Supplementary 7.** The symbolic key for the kinds of interaction among rosmarinic acid and yeast protein receptors.
